# Duration of the posttraumatic confusional state after moderate and severe traumatic brain injury: impact of injury severity and preinjury factors

**DOI:** 10.3389/fneur.2026.1528488

**Published:** 2026-04-13

**Authors:** Camilla Sæterstad, Rabea Iris Pantelatos, Jonas Stenberg, Kent Gøran Moen, Asta Kristine Håberg, Anne Vik, Toril Skandsen

**Affiliations:** 1 Department of Neuromedicine and Movement Science, Faculty of Medicine and Health Sciences, Norwegian University of Science and Technology (NTNU), Trondheim, Norway; 2 Department of Clinical Sciences, Danderyd Hospital, Division of Rehabilitation Medicine, Karolinska Institutet, Stockholm, Sweden; 3 Department of Radiology and Nuclear Medicine, St. Olavs Hospital, Trondheim University Hospital, Trondheim, Norway; 4 Department of Circulation and Medical Imaging, Faculty of Medicine and Health Sciences, Norwegian University of Science and Technology (NTNU), Trondheim, Norway; 5 Department of Radiology, Vestre Viken Hospital Trust, Drammen Hospital, Drammen, Norway; 6 Department of Neurosurgery, St. Olavs Hospital, Trondheim University Hospital, Trondheim, Norway; 7 Department of Neuromedicine and Movement Science, NTNU MiDT National Research Center, SIMUT, St. Olav's University Hospital, Trondheim, Norway; 8 Clinic of Rehabilitation, St. Olavs Hospital, Trondheim University Hospital, Trondheim, Norway

**Keywords:** brain reserve, cognitive reserve, craniocerebral trauma, education, posttraumatic amnesia, posttraumatic confusional state

## Abstract

**Introduction:**

This study aimed to estimate the duration, of the posttraumatic confusional state (PTCS), and predictors of the duration after moderate and severe traumatic brain injury (TBI) in a prospective inception cohort.

**Materials and methods:**

In 424 surviving neurosurgical patients with moderate [Glasgow Coma Scale (GCS) score 9–13] or severe TBI (GCS score ≤ 8), PTCS duration was estimated from sources documenting confusion or amnesia. Associations between PTCS duration and age, sex, indices of injury severity, and proxies of cognitive (education) and brain reserve (preinjury brain-related disability) were analyzed using binary logistic regression.

**Results:**

The most common PTCS duration was ≤ 7 days in the moderate TBI group (58%) and >28 days in the severe TBI group (52%). In multivariable analyses, lower age (*p* < 0.001), higher education, higher GCS score (*p* < 0.001), lower Rotterdam CT score (*p* = 0.004–0.002) and no road traffic accident (*p* = 0.006) were associated with a PTCS duration ≤ 7 days. Higher age (*p* < 0.001), lower GCS score (*p* < 0.001), and higher Rotterdam CT score (*p* < 0.001) were associated with a PTCS duration >28 days. Proxies of brain reserve were not independently associated with PTCS duration.

**Conclusion:**

Cognitive reserve was associated with short, but not long, PTCS, while the proxy of brain reserve was not associated with PTCS. Age and injury-related variables were most consistently associated with PTCS duration. These results support the notion that PTCS is a foreseeable clinical phase after TBI, determined mainly by the brain injury itself.

## 1 Introduction

A state of confusion, usually temporary, is common after traumatic brain injury (TBI) of all severities. It is observed briefly in mild TBI, but for days, weeks, or even months in patients with moderate-to-severe TBI. This confusional state is characterized by disorientation, disturbances of attention, disrupted sleep-wake cycle, and in some cases, agitation. The level of confusion may fluctuate in severity (1). As the patient does not form continuous memories during this phase, anterograde amnesia is another characteristic feature (2). The pathophysiology of confusion following TBI is incompletely understood, but it is thought to involve structural brain changes alongside functional disruptions linked to inflammation and altered neurotransmitter activity (3, 4).

There is no universal agreement regarding the terminology that best describes this period of confusion and amnesia. The phase was initially labeled posttraumatic amnesia (PTA) (5), which was later suggested to be replaced by posttraumatic confusional state (PTCS) (6), to better capture the wide range of neurobehavioral features. Both terms are commonly used in the field of neurorehabilitation today (7). The term PTCS will be used in the following here. Moreover, the terms posttraumatic delirium, delirium associated with TBI, and delirium are also used in the literature (8), especially concerning patients with TBI in the intensive care unit (4, 9), typically studying the delirium as a disorder that was present or absent, and not as a phase of a certain duration.

The duration of PTCS was suggested as an index of head injury severity in 1961 (5) before the Glasgow Coma Scale (GCS) was published in 1974 (10). Later, a strong association between PTCS duration and the GCS score was found, as well as between other signs of injury severity (11). Accordingly, the duration of PTCS has been found to be a predictor of patient outcome (12–16).

Brain and cognitive reserve are two related concepts that refer to individual differences in resilience to brain pathologies. Brain reserve refers to the structural characteristics of the brain that may protect against the presence of brain pathology. While brain reserves can be described as preinjury hardware differences, cognitive reserve can be considered as software differences (17) allowing for cognitive performance that is better than expected considering other factors influencing the status of the brain (18). The protective effect of brain and cognitive reserve on pathology related to brain injury or other brain disease is often studied using proxies such as previous diseases (brain reserve) and education (cognitive reserve) (19–21). While brain and cognitive reserves are associated with risk of delirium in other medical conditions, less is known about the association between brain and cognitive reserve and PTCS. A previous study found that lower age and longer education were associated with shorter PTCS duration after TBI (11), suggesting that brain and cognitive reserves may affect the length of PTCS.

Importantly, most studies of PTCS have been conducted in rehabilitation centers because regular and frequent assessments of cognition with standardized tools are less feasible in acute hospital wards, especially outside the ICU. Therefore, the literature may be biased toward more severe cases of TBI.

The overall aim of this study was to determine the duration of PTCS in a comprehensive and consecutive prospective inception cohort of patients with moderate and severe TBI recruited in an acute neurosurgical setting. Furthermore, possible associations between PTCS duration and demographic variables, injury severity, and preinjury proxies of cognitive (education) and brain reserves (preinjury disability caused by disorders affecting the brain) were analyzed.

## 2 Materials and methods

### 2.1 Study setting

The study was conducted at St. Olavs Hospital, a level I/II trauma center that serves as a university hospital for all approximately 700,000 inhabitants in the Central Norway region, as well as a general hospital for around 300,000 within the region. The hospital admits most patients with moderate to severe TBI in the region (22).

### 2.2 Study participants and study procedures

**Figure 1:**
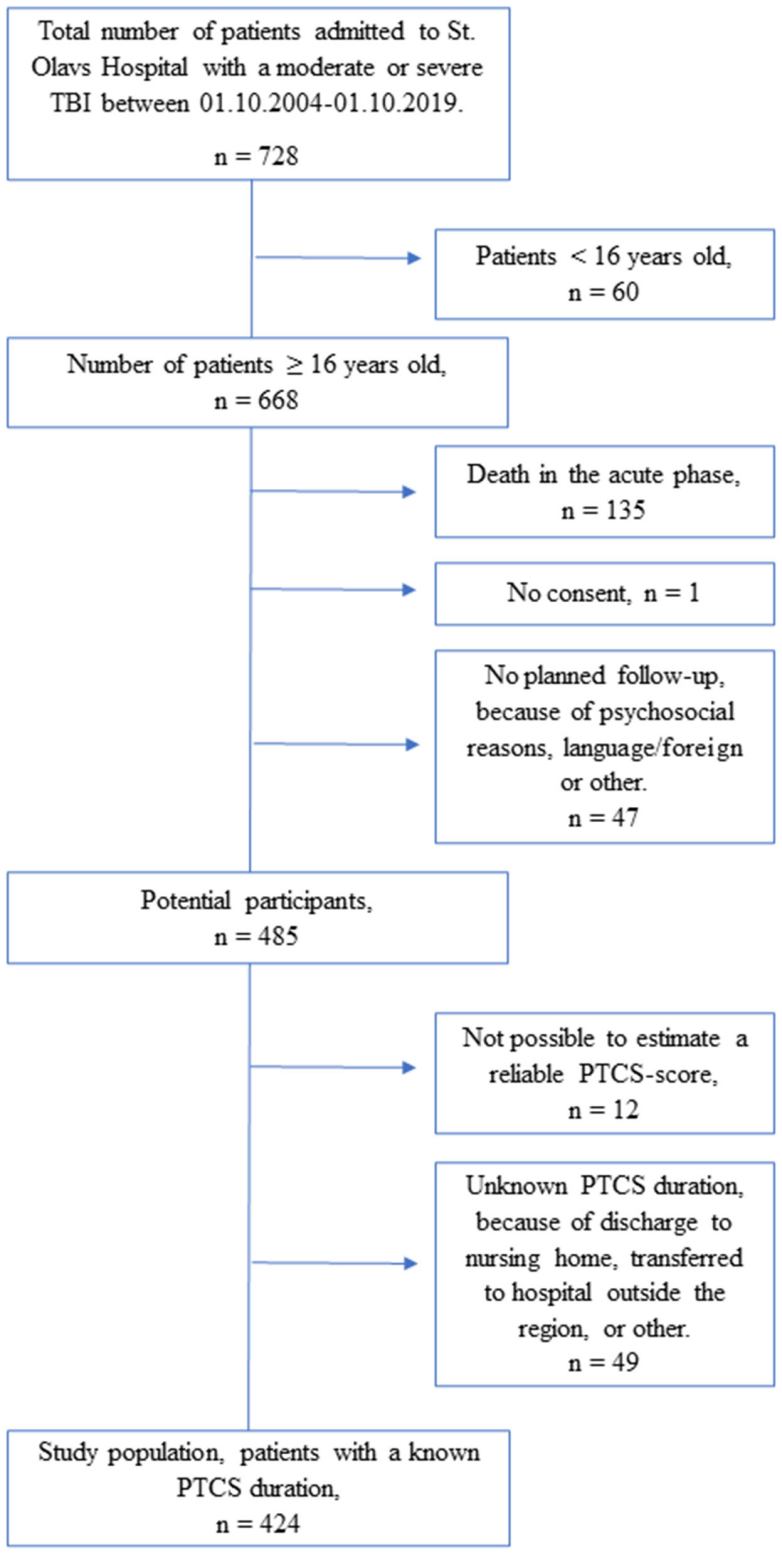
Flowchart showing included and excluded patients.

In total, 485 patients were screened for eligibility of the study (Figure 1). The final sample comprised 424 patients ≥16 years of age, with moderate (GCS score 9–13) or severe (GCS score ≤ 8) TBI, who had been prospectively included in the Trondheim moderate and severe TBI study between 01.10.2004 and 01.10.2019 and survived the acute hospital stay. Patients who lived in foreign countries, did not speak a Scandinavian language or English, or had severe psychosocial strains were excluded. A study nurse and residents in neurosurgery identified and included patients prospectively. Study personnel also checked the hospital's trauma registry regularly, and from 2015, a study nurse screened referrals to head CT scans weekly. Acute phase data were prospectively recorded.

### 2.3 Preinjury variables

Preinjury disabilities were categorized as “yes” if they affected daily life based on the judgement of the study personnel. Preinjury brain vulnerability served as a proxy for brain reserve and was categorized as “yes” when conditions considered to have a neurobiological substrate were present, such as alcohol abuse, narcotic abuse, psychiatric conditions, neurological diseases, developmental disorders, and multiple or unclassified disabilities.

### 2.4 Injury-related variables

The GCS score (3–13) at hospital admission was used; in cases of prehospital intubation, the lowest non-sedated GCS score was used. The GCS score was considered unreliable and handled as missing in cases of severe intoxication, sedation, and hypotension/hypoxia and as missing if no GCS score was recorded before sedation (*n* = 19). In cases where the GCS score was unreliable and no severity category of moderate or severe TBI could be determined, patients were excluded from the study population.

CT head findings were reviewed specifically for this database by radiologists and residents in radiology and neurosurgery, blinded to patient outcome, and the worst CT findings were described according to the specific lesions and used for scoring according to the Rotterdam CT classification (23).

### 2.5 PTCS estimation

In patients transferred to specialized rehabilitation during PTCS, the duration of PTCS was predominantly assessed prospectively with validated measures such as the Galveston Orientation and Amnesia Test (24) or the Orientation Log (25). In the majority of the remaining cases, PTCS duration was retrospectively estimated by reviewing medical records by CS and RIP under TS supervision. All available information was used, for example, notes made by healthcare professionals while the patient was hospitalized, including information from patients and their relatives describing the patient's condition, and documented retrospective evaluations performed at the standardized 6 months follow-up of all patients (26). Disorientation for time, place and situation and whether the patient was confused and agitated was evaluated. The documented return of day-to-day memory served as a marker of PTCS resolution. The PTCS duration was stratified into weekly intervals ( ≤ 7, 8–14, 15–21, 22–28 and >28 days) (27). The reason for this categorization was that in many cases the notes in the medical records were not sufficiently frequent and precise to pinpoint the exact day a patient was out of PTCS, while it was mostly possible to determine the patients' PTCS duration in weekly intervals. A PTCS duration interval was assigned to 424 patients out of the 485 potential study participants (87%). In 12 patients, a reliable PTCS duration interval could not be estimated, mostly because they had been sedated, due to the TBI or other injuries, and were oriented when sedation was ceased. In 49 patients, the PTCS duration was unknown, mostly due to transfer to a nursing home or a hospital outside the region while still in PTCS (Figure 1).

### 2.6 Statistical analyses

Histograms showed that age and GCS score were non-normally distributed, and the non-parametric Independent-samples Jonckheere–Terstra Test for ordered alternatives was used to compare age, GCS score, and Rotterdam CT score between the PTCS duration groups. For the comparison of proportions, we used the Pearson's chi-square test. An overview of the different types of TBI-related CT findings is provided. In the statistical analyses, the Rotterdam CT score was used.

Univariable and multivariable binary logistic regression analyses were used to analyze the association between the categorical PTCS duration and the explanatory variables. Listwise deletion was applied to missing data. External cause of injury was dichotomized into RTA, yes or no. The Rotterdam CT scores were recoded into three categories (1–2, 3–4, and 5–6). The dependent variable, PTCS, was dichotomized into two levels in two separate regression analyses. First, PTCS duration was dichotomized into ≤ 7 vs. >7 days, and second, into ≤ 28 vs. >28 days. Binary logistic regression was chosen because the assumption of proportional odds for ordinal regression was violated. Since most of patients had either a PTCS duration of ≤ 7 or >28 days (Table 1) two different cutoff values were applied. Interactions between preinjury and injury-related variables (e.g., testing whether the effect of age, education, and preinjury brain vulnerability on PTCS depended on Rotterdam CT scores) were investigated, omitting the interaction terms from the statistical models if they were not statistically significant.

**Table 1:** Patient demographics, injury-related factors and proxies of preinjury cognitive and brain reserves in relation to PTCS duration.

Variables	PTCS duration					*p* value
	0–7 days (*n* = 171)	8–14 days (*n* = 51)	15–21 days (*n* = 29)	22–28 days (*n* = 38)	>28 days (*n* = 135)	
Age, median [IQR]	38.5 [23.5–54.8]	43.0 [24.7–61.1]	39.9 [23.3–52.9]	47.2 [33.3–58.9]	49.6 [26.0–64.9]	0.02^a^
Sex, *n* (%) men	121 (71)	40 (78)	23 (79)	30 (79)	104 (77)	0.581^b^
Education^c^, *n* (%)						N.A.
Elementary school	27 (16)	8 (16)	7 (24)	8 (21)	35 (26)	
US Vocational training	52 (30)	23 (45)	14 (48)	14 (37)	44 (33)	
US General studies	25 (15)	3 (6)	2 (7)	4 (11)	10 (7)	
Bachelor	31 (18)	9 (13)	3 (10)	6 (16)	19 (14)	
Master	26 (15)	3 (6)	2 (7)	4 (11)	5 (4)	
Unknown	10 (6)	5 (10)	1 (3)	2 (5)	22 (16)	
Preinjury brain vulnerability, *n* (%)^d^						
Yes	25 (15)	9 (18)	7 (24)	6 (16)	34 (25)	0.169^b^
RTA, *n* (%)	65 (38)	25 (49)	17 (59)	20 (53)	64 (47)	0.131^b^
GCS score, median [IQR]	12 [9–13]	10 [6.5–12]	9 [7–12.75]	8 [4–11]	6 [4–9]	<0.001^e^
Worst Rotterdam CT score, *n* (%)						<0.001^a^
1	11 (6)	1 (2)	0 (0)	0 (0)	2 (2)	
2	68 (40)	15 (29)	11 (38)	11 (29)	15 (13)	
3	76 (44)	26 (51)	13 (45)	15 (40)	53 (39)	
4	14 (1)	7 (14)	1 (3)	6 (16)	38 (28)	
5	2 (2)	1 (2)	2 (7)	6 (16)	21 (16)	
6	0 (0)	1 (2)	2 (7)	0 (0)	6 (4)	
CT findings, *n* (%)						
Skull fracture	84 (49)	25 (49)	14 (48)	22 (58)	95 (70)	
tSAH	84 (49)	33 (65)	17 (59)	27 (71)	110 (82)	
SDH	68 (40)	26 (51)	12 (41)	24 (63)	91 (67)	
Epidural hematoma	39 (23)	4 (8)	3 (10)	7 (18)	19 (14)	
Single cortical contusion	30 (18)	9 (18)	6 (21)	5 (13)	13 (10)	
Multiple cortical contusions	55 (32)	22 (43)	11 (38)	23 (61)	90 (67)	
Evacuation of mass lesion	33 (19)	6 (12)	6 (21)	12 (32)	47 (35)	

GCS, Glasgow Coma Scale; IQR, interquartile range; N.A., not applicable; PTCS, posttraumatic confusional state; RTA, road traffic accidents; SD, standard deviation; SDH, subdural hematoma; tSAH, traumatic subarachnoid hemorrhage; US, upper secondary school.

^a^Independent-samples Jonckheere–Terstra Test for ordered Alternatives.

^b^Chi-Square Test.

^c^No p-value computed due to 24% of cells having expected count <5.

^d^Missing information about preinjury brain vulnerability of one patient.

^e^Missing information about GCS score of 19 patients.

Data were collected using a web-based data collection system administered by the Faculty of Medicine and Health Sciences at the Norwegian University of Science and Technology (NTNU), Trondheim, Norway. All analyses were conducted using the IBM SPSS version 29. A *p* value of <0.05 was considered statistically significant.

## 3 Results

### 3.1 Characteristics of the study population

The study population comprised 424 patients: 238 patients with moderate TBI and 186 patients with severe TBI. The mean age at injury was 43.8 years, 75% were men, and most had no university degree (72%). Preinjury brain vulnerability was present in 19% of the patients (Tables 2, 3). The most common external causes of injury were road traffic accidents RTA (45%) and falls (44%). Skull fractures (57%), traumatic subarachnoid hemorrhage tSAH (64%), subdural hematoma SDH (52%), and multiple cortical contusions (47%) were the most common CT findings (Table 3).

**Table 2:** Demographic characteristics of the 424 patients in the study population.

Variable	Included patients, *n* = 424
Age at injury, years	
Mean (SD)	43.8 (19.7)
Median [IQR]	43.3 [24.8–60.5]
Sex, *n* (%)	
Male	318 (75.0)
Education before injury, *n* (%)^a^	
Elementary school	85 (22.1)
Upper secondary school, vocational training	147 (38.3)
Upper secondary school, general studies	44 (11.5)
University bachelor ( ≤ 3 years)	68 (17.7)
University masters or higher (>3 years)	40 (10.4)
Missing	40 (9.4)
Preinjury disabilities, *n* (%)^a^	
None	339 (80.1)
Alcohol abuse	26 (6.1)
Narcotics abuse	5 (1.2)
Psychiatric conditions	11 (2.6)
Neurological diseases	15 (3.5)
Developmental disorders	3 (0.7)
Multiple	3 (0.7)
Other	3 (0.7)
Yes, but unclassified	18 (4.3)
Missing	1 (0.2)
Preinjury brain vulnerability, *n* (%)^a^	
Yes	81 (19.1)
No	342 (80.9)
Missing	1 (0.2)

IQR, interquartile range; SD, standard deviation.

^a^The categories' percentages are based on the number of patients with a valid value.

**Table 3:** Injury-related characteristics of the 424 patients in the study population.

Variable	Included patients, *n* = 424
External cause of injury, *n* (%)	
Road traffic accident	191 (45.0)
Fall	186 (43.9)
Assault	15 (3.5)
Other	24 (5.6)
Unknown	8 (1.9)
Severity of TBI	
GCS score, median [IQR]^a^	9.0 [6.0–13.0]
Moderate TBI (GCS score 9–13), *n* (%)	238 (56.1)
Severe TBI (GCS score 3–8), *n* (%)	186 (43.9)
CT findings, *n* (%)	
Skull fracture	240 (56.6)
Traumatic subarachnoid hemorrhage	271 (63.9)
Subdural hematoma	221 (52.1)
Epidural hematoma	72 (17.0)
Single cortical contusion	63 (14.9)
Multiple cortical contusions	201 (47.4)
Evacuation of mass lesion	104 (24.5)
Rotterdam CT score (worst)	
1	14 (3.3)
2	120 (28.3)
3	183 (43.2)
4	66 (15.6)
5	32 (7.5)
6	9 (2.1)
Median [IQR]	3.0 [2.0–4.0]

GCS, Glasgow Coma Scale; IQR, interquartile range; SD, standard deviation; TBI, traumatic brain injury.

^a^GCS at admission or preintubation. In 19 patients GCS score was missing.

**Figure 2:**
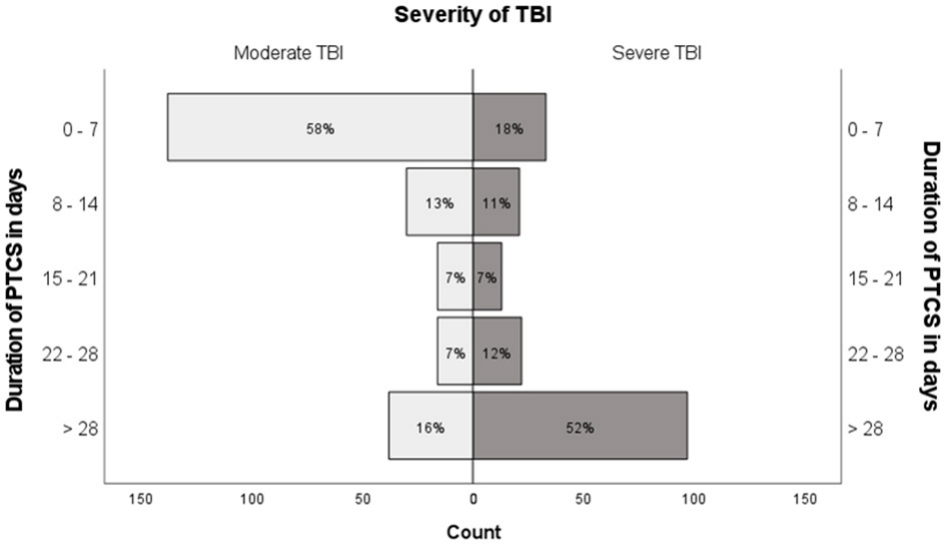
Distribution of PTCS duration in moderate and severe traumatic brain injury.

In patients with moderate TBI, a PTCS duration ≤ 7 days was the most common (58%); in severe TBI, a PTCS duration >28 days (52%). However, 16% of patients with moderate TBI had a PTCS duration of >28 days and 18% of patients with severe TBI had a PTCS duration of ≤ 7 days (Figure 2).

### 3.2 Differences between the PTCS duration groups

Age was significantly different between the PTCS duration groups (Table 1). The highest age was observed in the two groups with the longest PTCS duration. Being a man, having a university degree, preinjury brain vulnerability, or being injured in an RTA did not differ significantly between the PTCS duration groups. The GCS score was highest in the group with the shortest PTCS duration and decreased significantly with longer PTCS duration. The most severe CT findings and higher Rotterdam CT scores were more common when the PTCS duration increased.

### 3.3 Prediction of PTCS duration ≤ 7 days

In the univariable and multivariable analyses, lower age, higher GCS score, lower Rotterdam CT score, and not being in an RTA and having upper secondary school preparing for general studies, or a master's degree were significantly associated with a PTCS duration of ≤ 7 days (Table 4).

**Table 4:** Factors associated with PTCS duration ≤ 7 days.

Variable	All patients *n* = 368 *n* (%)	PTCS ≤ 7 days *n* = 156 *n* (%)	OR_crude_ (95% CI)	*p* value (OR_crude_)	OR_adj_ (95% CI)	*p* value (OR_adj_)
Age, years	42.6	39.7	0.99 (0.98–1.00)	0.015	0.96 (0.95–0.98)	<0.001
Sex						
Women	91 (24.7)	46 (29.5)	Ref.		Ref.	
Men	277 (75.3)	110 (70.5)	0.64 (0.40–1.04)	0.070	0.61 (0.32–1.16)	0.134
Education before injury				0.001		0.030
Elementary school	82 (22.3)	27 (17.3)	Ref.		Ref.	
US Vocational training	143 (38.9)	50 (32.1)	1.10 (0.62–1.95)	0.756	0.92 (0.44–1.92)	0.816
US General studies	43 (11.7)	25 (16.0)	2.83 (1.32–6.06)	0.007	2.84 (1.05–7.65)	0.039
Bachelor	63 (17.1)	30 (19.2)	1.85 (0.94–3.64)	0.074	1.42 (0.61–3.32)	0.415
Master	37 (10.1)	24 (15.4)	3.76 (1.66–8.51)	0.001	2.93 (1.09–7.85)	0.033
Preinjury brain vulnerability						
No	311 (84.5)	137 (87.8)	Ref.		Ref.	
Yes	57 (15.5)	19 (12.2)	0.64 (0.35–1.15)	0.134	0.74 (0.35–1.59)	0.447
RTA						
No	202 (54.9)	96 (61.5)	Ref.		Ref.	
Yes	166 (45.1)	60 (38.5)	0.63 (0.41–0.95)	0.028	0.38 (0.21–0.68)	0.001
GCS score (median)	10	12	1.38	<0.001	1.43 (1.30–1.57)	<0.001
			(1.28–1.50)			
Worst Rotterdam CT score				<0.001		<0.001
1–2	122 (33.2)	73 (46.8)	Ref		Ref	
3–4	211 (57.3)	81 (51.9)	0.42 (0.27–0.66)	<0.001	0.42 (0.23–0.76)	0.004
5–6	35 (9.5)	2 (1.3)	0.04 (0.01–0.18)	<0.001	0.06 (0.01–0.35)	<0.001

GCS, Glasgow Coma Scale; OR, odds ratio; RTA, road traffic accidents; US, upper secondary school.

### 3.4 Prediction of PTCS duration >28 days

In the univariable and multivariable analyses, older age, lower GCS score, and higher Rotterdam CT score were significantly associated with a PTCS duration of >28 days. Education and preinjury brain vulnerability were only significantly associated with PTCS duration in the univariable analysis (Table 5).

**Table 5:** Factors associated with PTCS duration >28 days.

Variable	All patients *n* = 368 *n* (%)	PTCS >28 days *n* = 107 *n* (%)	OR_crude_ (95% CI)	*p* value (OR_crude_)	OR_adj_ (95% CI)	*p* value (OR_adj_)
Age, years	42.6	46.8	1.02 (1.00–1.03)	0.008	1.03 (1.02–1.05)	<0.001
Sex						
Women	91 (24.7)	24 (22.4)	Ref.		Ref.	
Men	277 (75.3)	83 (77.6)	1.19 (0.70–2.03)	0.513	0.98 (0.49–1.96)	0.960
Education before injury				0.023		0.21
Elementary school	82 (22.3)	34 (31.8)	Ref.		Ref.	
US Vocational training	143 (38.9)	42 (39.3)	0.59 (0.33–1.04)	0.066	0.69 (0.33–1.43)	0.314
US General studies	43 (11.7)	9 (8.4)	0.37 (0.16–0.88)	0.024	0.34 (0.12–0.98)	0.045
Bachelor	63 (17.1)	17 (15.9)	0.52 (0.26–1.06)	0.072	0.70 (0.29–1.66)	0.415
Master	37 (10.1)	5 (4.7)	0.22 (0.08–0.62)	0.004	0.32 (0.09–1.11)	0.072
Preinjury brain vulnerability						
No	311 (84.5)	83 (77.6)	Ref.		Ref.	
Yes	57 (15.5)	24 (22.4)	2.00 (1.12–3.58)	0.020	1.73 (0.80–3.70)	0.162
RTA						
No	202 (54.9)	60 (56.1)	Ref.		Ref.	
42.6-2.2,-10.3498ptYes	166 (45.1)	47 (43.9)	0.94 (0.59–1.47)	0.770	1.19 (0.64–2.21)	0.577
GCS score (median)	10	6	0.74	<0.001	0.68	<0.001
			(0.69–0.80)		(0.62–0.76)	
Worst Rotterdam CT score				<0.001		<0.001
1–2	122 (33.2)	14 (13.1)	Ref		Ref	
3–4	211 (57.3)	71 (66.4)	3.91 (2.09–7.31)	<0.001	3.75 (1.78–7.89)	<0.001
5–6	35 (9.5)	22 (20.6)	13.06 (5.40–31.58)	<0.001	5.05 (1.82–14.07)	0.002

GCS, Glasgow Coma Scale; OR, odds ratio; RTA, road traffic accidents; US, upper secondary school.

## 4 Discussion

In this neurosurgical inception cohort of patients with moderate or severe TBI, more than half of the patients with moderate TBI had a PTCS duration ≤ 7 days, while few (18%) with severe TBI had PTCS this short, and approximately half had a PTCS duration interval >28 days. In multivariable analyses, older age and higher injury severity were strongly associated with a longer duration of PTCS. The proxies of cognitive and brain reserve (i.e., education and preinjury brain vulnerability) showed inconsistent and weak associations with PTCS.

### 4.1 The duration of PTCS and injury severity

PTCS duration was clearly associated with injury severity, as the shortest PTCS interval was most common in moderate TBI, the longest PTCS interval was most common in severe TBI, and GCS score and Rotterdam CT score were significantly independently associated with the duration of PTCS in all models. There is limited research on PTCS duration in patients with moderate TBI, but our study extends previous findings in a small study of 67 patients under 65 years of age who were admitted with a GCS score of 9–14 and PTCS of at least 1 day, with a mean duration of PTCS of 7 days (29).

Even though more than half of the patients with moderate TBI in our study had a PTCS duration of ≤ 7 days, and half of the patients with severe TBIs had a duration of >28 days, patients with both moderate and severe TBI experienced all the different PTCS intervals, as shown in Figure 2. This finding suggests that an estimate of the duration of PTCS could supplement the GCS in severity classification in the clinic and be useful in settings and studies where GCS scores are not available.

Not being injured in a RTA, a proxy for a lower burden of traumatic axonal injury (TAI) (28), was associated with a PTCS duration of ≤ 7 days in this study. In an MRI study of a subgroup of the current study sample, we have previously shown an association between the TAI severity and prolonged PTCS (26). Little is known about the pathophysiology of PTCS, but a functional disconnection between brain networks has been suggested (31).

### 4.2 Duration of PTCS and the role of age and cognitive and brain reserves

Age was the preinjury variable showing the strongest and most consistent independent association with PTCS, although a wide age span was observed in all PTCS duration groups. Higher age was associated with a PTCS duration of >28 days, and lower age was associated with a PTCS duration of ≤ 7 days in all analyses, also when adjusting for injury severity. Thus, our study adds to the literature by demonstrating the importance of age for PTCS duration. This relationship is likely explained by the negative effects of aging on brain resilience (18), in line with studies demonstrating the increased risk of delirium in older adults with TBI (9, 32).

Our proxy of brain reserve, presence of preinjury brain vulnerability, was associated with PTCS duration >28 days, but only in the univariable analysis; however, the OR was relatively unchanged in the multivariable model. A true effect may thus exist. Taken together, since age is associated with PTCS, and age is related to brain reserve (19), brain reserve and brain resilience seem to play a role in PTCS, which encourages further studies.

Cognitive reserve seemed to be associated with PTCS. This link was most notable for the PTCS duration ≤ 7 group, but a similar trend was present in the PTCS duration >28 days, although with ORs closer to 1. This suggests that the link between cognitive reserve and PTCS is weaker in people with TBI and long duration of PTCS, where injury severity contributes more to PTCS duration. The number of cases in some of the educational groups was, however, lower in the PTCS duration >28 days, in particular in the master's degree group, affecting the precision of the estimates. This could explain the discrepancy between our results and the consistent association between PTCS duration and education in another study in young patients admitted for inpatient rehabilitation (11). Education was also found to attenuate the negative effects of moderate-to-severe TBI on cognition, as assessed by neuropsychological tests in a previous study (21), further supporting a predictive role of cognitive reserve.

### 4.3 The main determinants of PTCS duration

It seems clear that injury severity (injury mechanisms, GCS score, and head CT findings) had the strongest impact on PTCS duration in this study. This finding supports the view that PTCS is a distinct and expected clinical phase after moderate and severe TBI and is mostly determined by the head trauma itself. Although some clinical symptoms of PTCS and likely certain pathophysiological aspects resemble those observed in delirium among other patient groups, and recent research has explored potential common biomarkers (3), our study showed that there are also differences. While delirium in critical illness, infections, or other systemic diseases occurs predominantly in older patients or other patients who are frail and have preinjury cognitive impairments, PTCS is observed in almost *all* patients with moderate or severe TBI, at all ages. Furthermore, PTCS often lasts for weeks in severe TBI, while delirium in the ICU in patients without brain injury tends to last only a few days (30, 33, 34).

### 4.4 Strengths and limitations

Strengths were that patients were recruited from the time of injury, and the high rate of inclusion. Limitations were, first, that we lacked accurate and prospectively collected information about PTCS duration in many cases, which was to be expected in an acute hospital setting. Therefore, PTCS was estimated retrospectively based on information from medical records. Second, data was lacking for patients who were transferred to other facilities outside the health region while still in PTCS. Third, the categorization of preinjury disability was coarse. For preinjury disability to be categorized as present, it had to affect the daily life of the patient based on the judgement of healthcare personnel. Additionally, some inter-rater variability could be present. Finally, study findings need to be validated in other settings. In the present study, only patients admitted to a level I/II trauma center were included, and findings may not generalize to patients who are treated at their local hospitals, that is some patients with moderate TBI and older patients (22). Future studies should examine the proposed determinants of PTCS duration using larger, multicenter datasets, such as the TRACK-TBI cohort.

### 4.5 Conclusion

In this neurosurgical cohort of moderate and severe TBI, PTCS duration ≤ 7 days was found in more than half of the moderate TBI cases, while in severe TBI, few had ≤ 7 days of PTCS, and >28 days was found in half of the patients. A pragmatic estimation of the duration of PTCS seems feasible as a supplement to the GCS score and should be further studied. The PTCS duration was independently associated with all indices of brain injury severity and age. The strong impact of brain injury severity on PTCS duration and the, at best, modest association between PTCS duration and cognitive and brain reserves indicates that PTCS is determined mainly by the brain injury itself and should not be regarded as a delirium in particularly vulnerable individuals.

## Data Availability

The datasets presented in this article are not publicly available because national legislation prohibits open sharing. However, the data may be provided upon request, subject to the establishment of a formal collaboration agreement and receipt of the required approvals under applicable national regulations. Requests to access the datasets should be directed to Toril Skandsen, toril.skandsen@ntnu.no.
